# Treatment of Community-Acquired Pneumonia: A Case Report and Current Treatment Dilemmas

**DOI:** 10.1155/2017/5045087

**Published:** 2017-06-15

**Authors:** Glenn Harnett

**Affiliations:** No Resistance Consulting Group, Mountain Brook, AL, USA

## Abstract

Resistance to macrolides is rising in the USA and warrants careful consideration when confronted with a patient with suspected pneumonia in the urgent care clinic. This case study exemplifies the potentially serious consequences of treatment failure following prescription of a macrolide for community-acquired bacterial pneumonia. Furthermore, the consequential treatment dilemmas currently faced by physicians are briefly discussed.

## 1. Introduction

Each year, over 4 million ambulatory patients are treated for community-acquired pneumonia (CAP) in the United States (US) [1], with approximately 80% treated on an outpatient basis [2]. Community-acquired bacterial pneumonia (CABP) is a common presenting illness in the urgent care setting, yet many providers underappreciate the mortality associated with pneumonia and underrate how commonly it occurs—CABP is in fact the leading cause of infectious death in adults and the number of deaths is higher than either breast or prostate cancer [3].

This case report describes common historical and physical examination findings in CABP and the use of traditional and more modern diagnostic tools, as well as treatment dilemmas currently facing clinicians.

*Streptococcus pneumoniae* remains the leading bacterial cause of pneumonia in the United States and globally. Moreover, of particular concern to public health agencies and clinicians is that* S. pneumoniae* is rapidly becoming more resistant to currently available antibiotics, elevating to prominence new phenotype serotypes referred to as drug-resistant* S. pneumoniae* (DRSP).

These DRSP serotypes are particularly resistant to currently available macrolides, such as azithromycin.* S. pneumoniae* macrolide resistance rates are as high as 60% or more in some regions of the US [1]. The current IDSA/ATS guidelines on the management of CAP (soon to be updated) recommend the use of an alternative to macrolides in areas where “high-level” (minimum inhibitory concentration [MIC] ≥ 16 *µ*g/mL) macrolide-resistant* S. pneumoniae* rates are greater than 25% [4]. Keep in mind that presently those areas include the entire US, other than the CDC defined mountain region [5]. Despite this, macrolides are used to treat approximately 40% of CABP cases in the US [6].

Clinicians should also be aware of the correlation between pneumonia and influenza. Influenza is a predisposing factor for acquiring pneumonia, especially in older adults and those with comorbid conditions (see later). Indeed, pneumonia is the most common significant complication of influenza and leads to significant morbidity and mortality.

## 2. Case Presentation

A 66-year-old male presented to an urgent care clinic with a 4-day history of dry cough, progressing to rusty colored sputum, sudden onset of chills the previous evening, subjective fever, and malaise. Originally, the man thought he had a cold, but the symptoms had worsened and he “barely slept last night with all this coughing.”

He denied experiencing shortness of breath but suggested he may be breathing “a little faster than normal.” He related that, on the way to the clinic, he felt some sharp right-sided chest pain after a particularly long bout of coughing. He denied any leg swelling, orthopnea, or left-sided/substernal chest pain. He also denied any gastrointestinal symptoms (no nausea, vomiting, or diarrhea). His past medical history included hypertension and hypercholesterolemia. He reported no antibiotic use in the previous three months.

He was anxious to “get something to clear this up” as he had plans to attend his first granddaughter's destination-wedding in the Caribbean in one week's time.

## 3. Physical Examination

In general, the man appeared tired and a bit “washed out.” His vital signs were as follows:

- Temperature (F): 101.3
- Blood pressure (mmHg): 128/76
- HR (bpm): 102
- RR (bpm): 24
- SpO_2_ (%): 94

Respiratory examination revealed mild tachypnea with dullness to percussion over the lower-right lung. Auscultation revealed decreased breath sounds in the same area, but no crackles or wheezing.

Other than mild tachycardia with a regular rhythm, the remainder of the physical examination was normal. There was no jugular venous distention or pedal edema. For comparison and consideration, other theoretical physical examination findings that would have been indicative of pneumonia are presented in Table 1 [7], and the differential diagnosis is in Table 2.

## 4. Diagnostic Results

**Figure 1:**
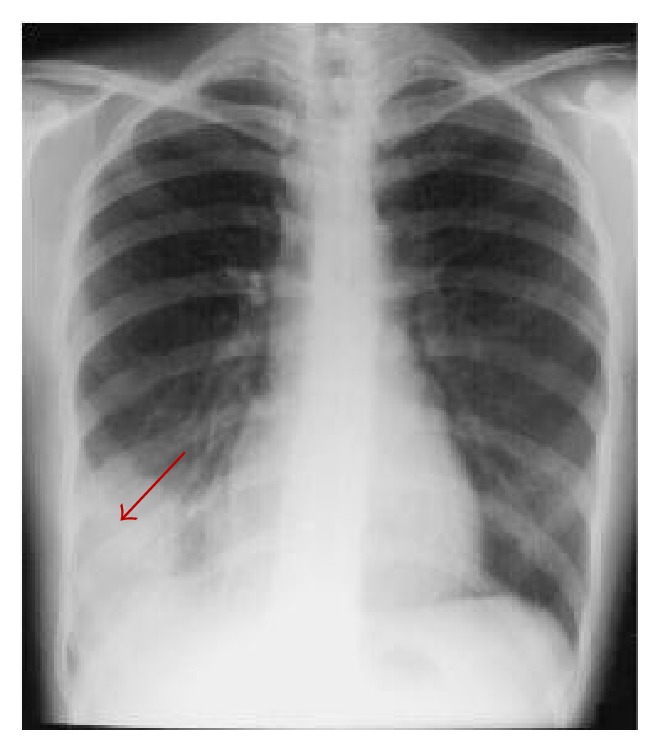
Example of lower-right lobar shadow (red arrow) from a representative PA radiograph.

The “gold standard” for diagnosis of CABP is the chest X-ray. When pneumonia is suspected based on history of present illness, subjective symptoms, and physical exam, the clinician should obtain a standard chest radiograph with PA and lateral views. The chest X-ray can also be helpful in “ruling out” other potential causes of symptoms, even if infiltrates may not always be visible to confirm CABP with some early presentations of CABP. The man's chest X-ray revealed a lower-right lobar-type pneumonia without an effusion (Figure 1).

Table 3 shows selected results from the man's complete blood-cell count (CBC) and complete metabolic panel (CMP). Note that the patient's WBC (4,200 cells/uL) and percentage of lymphocytes (12%) was lower than normal (18–40%).

Although not done prior to initiating treatment in this case, other testing options may have included blood cultures, urine antigen testing for* S. pneumoniae* and Legionella, and sputum cultures. The vast majority of urgent care centers do not have the capability of performing blood cultures or collecting sputum samples, nor do many, at this point, routinely collect urine antigen samples in patients with presumed pneumonia.

## 5. Discussion

### 5.1. Risk Stratification

Initial risk stratification in CABP helps guide diagnosis, treatment decisions, and patient disposition. Hospital admission is an important economic consideration in CABP as the cost of inpatient care for pneumonia is logarithmically higher than outpatient care (e.g., circa $27k versus $2k per episode, resp.) [9, 10].

Moreover, low risk CABP patients ought to be treated as outpatients whenever possible to avoid complications of hospital-acquired superinfections and thromboembolic events [11]. CABP patients treated on an outpatient basis are also more likely to return to work and other activities faster than those admitted, while most patients prefer to be treated as an outpatient [12].

Providers making site-of-care treatment decisions need to consider barriers to outpatient treatment, such as frailty, lack of response to previous therapy, severe social or psychiatric problems, substance abuse, homelessness, and unstable living conditions.

Prognostic models, such as the PORT score (based on the Pneumonia Severity Index [PSI] scoring system), or severity-of-illness scores, such as the CURB-65 criteria, can aid the decision for outpatient treatment [4].

**Figure 2:**
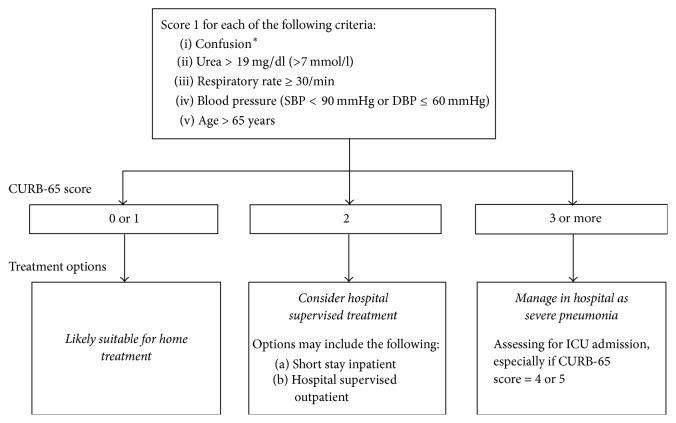
CURB-65 scoring: a simple, fast, and effective clinical decision tool for determining point of care setting in CAP. Urea/blood urea nitrogen (BUN) score can be excluded when unavailable in the urgent care setting. Figure adapted by authors from Lim et al., 2003, with permission [13]. ^*∗*^Defined as a Mental Test Score of 8 or less or new disorientation in person, place, or time.

The CURB-65 scale is a simple way to determine pneumonia severity. Using CURB-65, providers assign 1 point for each criterion met in Figure 2. If the individual scores 1 point or less, outpatient treatment is appropriate; 2 points indicate hospitalization and inpatient treatment. Greater than or equal to 3 points warrant inpatient treatment in the ICU [13]. In our patient's case, the CURB-65 score was 1, with the one point assigned based on his age of 66. He met none of the other CURB-65 criteria.

The use of the CURB-65 and PORT scores can be problematic in the urgent care setting as many centers do not have point of care chemistry testing and very few have access to arterial blood gas testing. However, even when tests are unavailable, the score for BUN can be excluded and if the patient still has a remaining CURB-65 score of 2 or higher, they clearly meet hospital admission criteria [13].

### 5.2. Pneumonia and Influenza

CABP together with influenza remains the 8th leading cause of death in the United States [14]. Between 1979 and 2009 there were an average of 66,000 deaths per year attributable to coinfection with influenza and pneumonia [3], with 55,227 deaths occurring in 2014 [14].* S. pneumoniae* is the leading cause of pneumonia in those coinfected with influenza and leads to higher morbidity and mortality. A common mistaken perception is that influenza itself has a high mortality rate. Complications account for the majority of morbidity/mortality in influenza, with pneumonia being the leading significant complication [15].

Historical review of the 1918-19 influenza pandemic suggests that the majority of deaths were not a direct effect of the influenza virus but instead resulted from bacterial coinfection causing pneumonia [15]. This remains true today [17], and for that reason, clinicians treating patients with influenza need to have a high clinical suspicion for pneumonia.

In patients with influenza, coinfection with bacterial pneumonia is something clinicians cannot afford to miss. Risk factors for bacterial pneumonia coinfection in influenza are listed in Table 4 [8]. Other influenza complications may include bacteremia, sepsis, empyema, pericarditis, respiratory failure, and death.

### 5.3. Treatment Options and Macrolide Resistance

The 2007 IDSA/ATS guidelines [4] recommend the antibiotic therapy options distilled in Table 5 for treatment of CAP. Guideline adherence and appropriate use of macrolides have been associated with reduced mortality in outpatients with pneumonia [18].

The guidelines provide detail not shown in Table 5, such as weighting of recommendations based on level of evidence, definitions, and examples of terms [4].

Approximately 40% of* S. Pneumoniae* isolates in the US display in vitro resistance to macrolide antibiotics. This resistance has developed via 2 separate mechanisms:

1. Mef(A)-mediated resistance involves an efflux pump, resulting in low-level resistance. High local concentrations of macrolide antibiotics can overcome this type of resistance mechanism, resulting in good clinical efficacy despite in vitro resistance.
2. Erm(B)-mediated resistance involves a conformational change to the macrolide binding site at the bacterial 23S ribosomal subunit. This change confers high-level macrolide resistance.

Two-thirds of macrolide resistance in the US is related to the mef(A) mechanism. However, erm(B)-mediated “high-level” resistance appears to be increasing, with the potential to lead to increased clinical treatment failures for patients treated with macrolide monotherapy [19].

**Figure 3:**
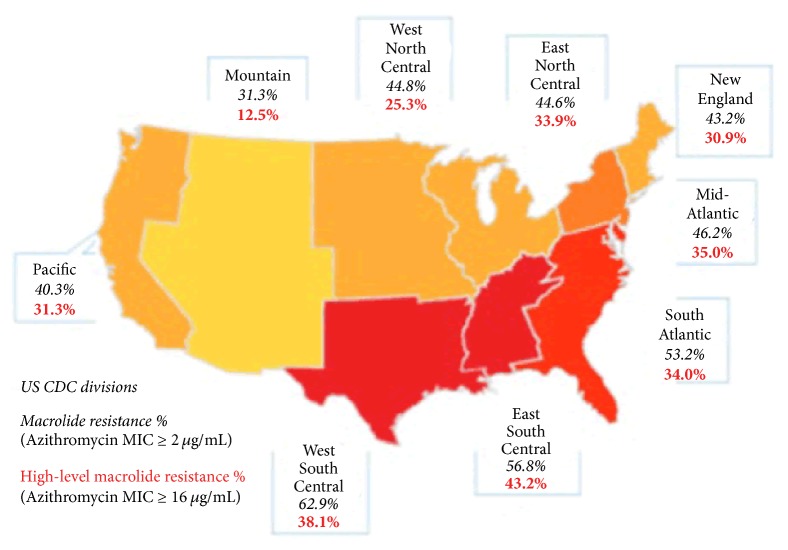
Rate of macrolide-resistant* S. pneumonia* in 2014. Figure adapted by authors from Blondeau and Theriault, 2017 [16].

*Important*. In regions with “high-level” (minimum inhibitory concentration [MIC] ≥ 16 *µ*g/mL) macrolide-resistant* S. pneumoniae*, consider the use of nonmacrolide alternative agents listed in Table 2, including those for patients without comorbidities [4]. Once again, keep in mind that only the mountain region of the USA has* S. pneumoniae “high-level” *(MIC ≥ 16 ug/ml)* macrolide* resistance rates lower than 25% (Figure 3), which means that most clinicians should reconsider the use of macrolides as monotherapy in CAP.

Along with local resistance rates, antibiotic selection should consider the patient's risk factors for possible infection with DRSP [5, 16, 20], including the following:

1. Recent antibiotic use (within 3 months)
2. Age greater than 65 years
3. Immunosuppressive illness
4. Multiple medical comorbidities
5. Exposure to a child attending a daycare center
6. Alcohol abuse
7. Asthma/COPD
8. Diabetes mellitus
9. Recent travel [5]

In the first decade of this millennium, DRSP risk factors were present in approximately half of outpatient CAP cases treated in the acute care setting. Despite this fact, physician adherence to guideline-concordant antibiotic therapy remained infrequent as clinicians continued to use macrolides, especially azithromycin, as CAP monotherapy [18]. This is despite the fact that guideline adherence and appropriate use of macrolides had been associated with reduced mortality in outpatients with pneumonia [18].

Remember that the most prevalent causative organism in CAP is* S. pneumoniae*, regardless of the host or setting. Empiric antibiotic therapy should always be selected with this microorganism in mind. The IDSA guidelines clearly recommend knowing the prevalence of high-level drug-resistant pneumococci in your geographic location to aid decision-making. Unfortunately, antibiograms are becoming less available to community physicians working outside the hospital setting. Healthcare leaders will need to work together to make these useful tools more available to clinicians as antibiotic resistant* E. coli*,* S. aureus*, and* S. pneumoniae* strains increasingly affect our patient population.

Response to antibiotic therapy for CABP should be evaluated within 48–72 hours of initiation of treatment. However, antibiotics should not be changed within the first 72 hours unless marked clinical deterioration occurs or the causative pathogen is identified. Chest X-rays usually clear within 4 weeks in patients younger than 50 years, but resolution may be delayed for 12 weeks or longer in older individuals. The benefit of routine radiography after pneumonia remains unclear. The most recent US guidelines do not address this issue, while a recent UK guideline recommends follow-up X-rays only for patients with persistent symptoms or those “at higher risk of underlying malignancy (especially smokers and those aged > 50 years)” [21].

## 6. Case Presentation: Patient Treatment Course and Outcome

Based on the patient's presentation and testing results, the patient was correctly diagnosed with CABP. Applying the CURB-65 criteria with a resultant score of 1, the man was appropriately treated on an outpatient basis. However, unaware that “high-level”* S. pneumoniae* macrolide resistance rates in the East South Central area are 48%, the provider placed the man on a “Z-PAK” (azithromycin) as CAP monotherapy.

Two days later, the man presented to the local ER with worsening symptoms that had progressed to include dyspnea and an oxygen saturation of 89%. He was admitted to the hospital for 5 days of inpatient treatment, including IV levofloxacin, with 2 days spent in the ICU. The patient did not require ventilator support. Blood cultures revealed* S. pneumoniae *resistant to azithromycin but sensitive to fluoroquinolones. The man survived his hospitalization.

Would further testing have changed the treatment plan or point of care decision? Blood cultures in CAP can be of questionable utility and are not routinely ordered in the outpatient setting. Obtaining blood cultures for non-ICU CAP patients is* no longer* core measure per CMS and JCAHO as of January 1, 2014. This is likely due to the fact that rates of positive blood cultures in confirmed CAP are only in the 8–15% range [20, 22]. Positive rates are even lower in those with low risk CAP. Even in pneumococcal pneumonia, the results are often negative (although their yield may be higher in patients with more severe pneumonia/infection) [23].

Per IDSA/ATS Consensus Guidelines,* S. pneumoniae* urine antigen testing (UAT) is suggested if testing results will change the antibiotic management for patients with CAP.* S. pneumoniae *UAT is an option currently available in labs that are certified as COLA/CLIA moderately complex. IDSA clinical indications for* S. pneumoniae* UAT testing in outpatients (which ought to be reimbursed) include [4] the following:

1. Failure of outpatient antibiotic therapy
2. Leukopenia
3. Active alcohol abuse
4. Severe liver disease
5. Asplenia
6. Pleural effusion
7. ICU admission

The man's CBC revealed that he was leukopenic and thereby met IDSA criteria for* S. pneumoniae *UAT testing. Considering the local antibiotic resistance rates, would a positive* S. pneumoniae* UAT test have changed the treatment plan in the man's case?

## 7. Considerations

Current IDSA/ATS guidelines recommend that in regions with a high rate (25%) of infection with high-level (MIC, ≥16 mg/mL) macrolide-resistant* S. pneumoniae*, macrolide monotherapy should be avoided [4, 24]. In the USA,* S. pneumoniae* resistance rates are increasing across antibiotic class [25], with* S. pneumoniae* even being fully resistant to one or more antibiotics in 30% of severe pneumonia cases [26]. High-level macrolide resistance to* S. pneumoniae* is increasing [26, 27], with many US states showing overall resistant rates greater than 40% (Figure 3) [16, 26].

Using the currently available macrolides as monotherapy in CABP should be reconsidered in this era of increased DSRP. Recently, Mandell suggested the increasing pneumococcal resistance to macrolides may diminish the use of these drugs as monotherapy for CAP [28]. Per the IDSA guidelines respiratory fluoroquinolones and doxycycline are the only other treatment considerations for monotherapy in CAP. A question that remains is how often high-level antibiotic resistance translates into actual treatment failure. Mandell points out that retrospective data show a positive correlation among macrolide resistance rates ≥ 25%, treatment failure, and costs [24]. Increased mortality in cases of CABP failing initial outpatient macrolide therapy was reported even with low-level macrolide resistance [29]. Other risks, besides treatment failure with macrolide monotherapy, includes the well documented “black box” side effects of the fluoroquinolones, such as tendinopathy, and their propensity for causing* C. difficile* enterocolitis [24]. Many infectious disease physicians worry that the fluoroquinolones are too broad spectrum for routine use in low risk outpatient treatment for CAP and that their use as monotherapy could lead to increased resistance in the future. Increasing antibiotic resistance rates to doxycycline have also limited its effectiveness as monotherapy in CAP [24].

This case illustrates the challenging outpatient treatment environment in which CABP resistance patterns have changed while our current arsenal of antibiotics has remained the same. Current awareness of resistance patterns is not ideal and providers need better access to local/regional information (antibiograms) and further education on preferred treatment options for CABP. New community-acquired pneumonia guidelines from the IDSA/ATS are expected in 2017. New antibiotics for the treatment of CAP are needed and hopes are that new regulatory processes such as those contained in Generating Antibiotic Incentives Now (GAIN) Act of 2012 will stimulate further antibiotic development. Another noteworthy CAP-related case you are encouraged to read was published by Aguilar et al., 2016 [30].

## Figures and Tables

**Table 1:** Physical examination findings in CAP [7].

(i) Adventitious breath sounds (rales/crackles, rhonchi, wheezes)
(ii) Decreased intensity of breath sounds
(iii) Dullness to percussion
(iv) Lymphadenopathy
(v) Pleural friction rub
(vi) Bradycardia^a^
(vii) Periodontal disease^b^
(viii) Bullous myringitis^c^
(ix) Cutaneous nodules^d^

a may indicate *Legionella* etiology; b may indicate an anaerobic and/or polymicrobial infection; c may indicate a *Mycoplasma* pneumonia infection; d may indicate a *Nocardia* infection via hematogenous spread from a pulmonary focus.

**Table 2:** Differential diagnosis in CAP.

If patient has concurrent chest pain, consider the following:
(i) MI
(ii) Tension pneumothorax
(iii) Esophageal rupture
(iv) Pericardial effusion
(v) Aortic dissection
(vi) Aortic aneurysm
(vii) Pulmonary embolus
(viii) Aspiration/pneumonitis
(ix) Atelectasis
(x) RSV/bronchiolitis
(xi) Acute bronchitis
(xii) COPD
(xiii) Foreign body aspiration
(xiv) Fungal pneumonia
(xv) Lung abscess
(xvi) PCP
(xvii) Respiratory failure
(xviii) Viral pneumonia
(xix) Neoplasm
(xx) Asthma

MI, myocardial infarction; RSV, respiratory syncytial virus; COPD, chronic obstructive pulmonary disease; PCP, *Pneumocystis jirovecii* pneumonia.

**Table 3:** Selected patient CBC and CMP results.

Blood-cell count	Liver-function test	Basic metabolic panel
WBC (cells/*µ*L): 4,200 (i) Neutrophils: 72% (a) Bands: 0% (ii) Lymphocytes: 12% Platelets (cells/ml): 180,000	Total protein (g/dL): 7.1 Albumin (g/dL): 3.9 to 5.0 Total bilirubin (mg/dL): 0.6 AST (IU/L): 18 ALT (IU/L): 23 AlkP (IU/L): 98	Sodium (mEq/L): 138 Potassium (mEq/L): 4.2 Chloride (mmol/L): 99 Calcium (mg/dL): 8 CO_2_ (/L): 28 BUN (mg/dL): 17 Creatinine (mg/dL): 1.1 Glucose (mg/dL): 87

AlkP, alkaline phosphatase; ALT, alanine aminotransferase; AST, aspartate aminotransferase; CBC, complete blood-cell count; CMP, complete metabolic panel; BUN, blood urea nitrogen; WBC, white blood cell.

**Table 4:** Influenza patients at greater risk of bacterial pneumonia [8].

(i) Adults > 65 years
(ii) Pregnant or postpartum (within 2 weeks after delivery) women
(iii) Persons ≤ 19 years receiving long-term aspirin therapy
(iv) American Indians and Alaska natives
(v) Morbidly obese (i.e., body mass index ≥ 40)
(vi) Residents of nursing homes and other chronic care facilities
(vii) Immunosuppressed persons
(viii) Chronic pulmonary (including asthma) disease
(ix) Renal, hepatic, and/or hematological (including sickle cell) disease
(x) Cardiovascular (except hypertension) disease
(xi) Metabolic disorders (including diabetes mellitus)
(xii) Neurologic and neurodevelopment conditions (including disorders for the brain, spinal cord, peripheral nerve and muscle, epilepsy, stroke, and intellectual disability [e.g., mental retardation])
(xiii) Moderate to severe development delay, muscular dystrophy, or spinal cord injury

**Table 5:** Summary^*∗*^ of 2007 IDSA/ATS guidelines for outpatient treatment of community-acquired pneumonia [4].

Conditions	Recommended treatment	Further detail
Region with **>25%** infection rate with “high-level” macrolide-resistant *S. pneumoniae*	Consider the nonmacrolide alternatives below	

Previously healthy *and* no risk factors for DRSP	Macrolide (preferred) or doxycycline	Macrolides: azithromycin, clarithromycin, or erythromycin

Comorbidities, *including the following*: (i) Recent use of antimicrobials (ii) Other risks for DRSP	Either respiratory fluoroquinolone or *β*-lactam *plus* macrolide (or doxycycline instead of macrolide)	Fluoroquinolones: moxifloxacin, gemifloxacin, or levofloxacin Preferred *β*-lactam: high-dose amoxicillin or amoxicillin-clavulanate

^*∗*^This distillation of recommendation is not intended to replace the guidelines, which contain details not shown here; DRSP, drug-resistant *S. pneumoniae*.

## References

[1] File T. M., Marrie T. J. (2010). Burden of community-acquired pneumonia in North American adults. *Postgraduate Medicine*.

[2] Mandell L. A. (2004). Epidemiology and etiology of community-acquired pneumonia. *Infectious Disease Clinics of North America*.

[3] Xu J., Murphy S. L., Kochanek K. D., Bastian B. A. (2016). Deaths: final data for 2013. *National Vital Statistics Reports*.

[4] Mandell L. A., Wunderink R. G., Anzueto A. (2007). Infectious diseases society of America/American thoracic society consensus guidelines on the management of community-acquired pneumonia in adults. *Clinical Infectious Diseases*.

[5] Tillotson G. S. (2016). Where in the world? The role of geography in antibiotic resistance and the potential impact in pulmonary infections. *Postgraduate Medicine*.

[6] Heath I. (2014). *National Disease and Therapeutic Index*.

[7] Kamangar N. (2016). Bacterial pneumonia clinical presentation. *Medscape: Drugs & Diseases*.

[8] Metersky M. L., Masterton R. G., Lode H., File T. M., Babinchak T. (2012). Epidemiology, microbiology, and treatment considerations for bacterial pneumonia complicating influenza. *International Journal of Infectious Diseases*.

[9] Broulette J., Yu H., Pyenson B., Iwasaki K., Sato R. (2013). The incidence rate and economic burden of community-acquired pneumonia in a working-age population. *American Health and Drug Benefits*.

[10] Sato R., Rey G. G., Nelson S., Pinsky B. (2013). Community-acquired pneumonia episode costs by age and risk in commercially insured US adults aged ≥50 years. *Applied Health Economics and Health Policy*.

[11] Niederman M. S., McCombs J. S., Unger A. N., Kumar A., Popovian R. (1998). The cost of treating community-acquired pneumonia. *Clinical Therapeutics*.

[12] Coley C. M., Li Y.-H., Medsger A. R. (1996). Preferences for home vs hospital care among low-risk patients with community-acquired pneumonia. *Archives of Internal Medicine*.

[13] Lim W. S., van der Eerden M. M., Laing R. (2003). Defining community acquired pneumonia severity on presentation to hospital: an international derivation and validation study. *Thorax*.

[14] Kenneth D., Kochanek M. A., Sherry L., Murphy B. S., Jiaquan Xu M. D., Betzaida Tejada-Vera M. S. (2016). Deaths: final data for 2014. *National Vital Statistics Reports*.

[15] Morens D. M., Taubenberger J. K., Fauci A. S. (2008). Predominant role of bacterial pneumonia as a cause of death in pandemic influenza: implications for pandemic influenza preparedness. *The Journal of Infectious Diseases*.

[16] Blondeau J. M., Theriault N. (2017). Application of the formula for rational antimicrobial therapy (FRAT) to community-acquired pneumonia. *Journal of Infectious Diseases & Therapy*.

[17] Joseph C., Togawa Y., Shindo N. (2013). Bacterial and viral infections associated with influenza. *Influenza and other Respiratory Viruses*.

[18] Asadi L., Eurich D. T., Gamble J.-M., Minhas-Sandhu J. K., Marrie T. J., Majumdar S. R. (2012). Guideline adherence and macrolides reduced mortality in outpatients with pneumonia. *Respiratory Medicine*.

[19] Feldman C., Anderson R. (2012). Antibiotic resistance of pathogens causing community-acquired pneumonia. *Seminars in Respiratory and Critical Care Medicine*.

[20] Yu V. L., Chiou C. C. C., Feldman C. (2003). An international prospective study of pneumococcal bacteremia: correlation with in vitro resistance, antibiotics administered, and clinical outcome. *Clinical Infectious Diseases*.

[21] Lim W. S., Baudouin S. V., George R. C. (2009). British Thoracic Society guidelines for the management of community acquired pneumonia in adults: update 2009. *Thorax*.

[22] Makam A. N., Auerbach A. D., Steinman M. A. (2014). Blood culture use in the emergency department in patients hospitalized for community-acquired pneumonia. *JAMA Internal Medicine*.

[23] Luna H. I. R., Pankey G. (2001). The utility of blood culture in patients with community-acquired pneumonia. *The Ochsner Journal*.

[24] Asche C., McAdam-Marx C., Seal B., Crookston B., Mullins C. D. (2008). Treatment costs associated with community-acquired pneumonia by community level of antimicrobial resistance. *Journal of Antimicrobial Chemotherapy*.

[25] Flamm R. K. Activity of omadacycline tested against Streptococcus pneumoniae from a global surveillance program.

[26] CDC Report Antibiotic resistance threats in the United States.

[27] Center for Disease Dynamics EaPC Macrolide-resistant Streptococcus pneumoniae.

[28] Mandell L. A. (2016). Something new for community-acquired pneumonia?. *Clinical Infectious Diseases*.

[29] Iannini P. B., Paladino J. A., Lavin B., Singer M. E., Schentag J. J. (2007). A case series of macrolide treatment failures in community acquired pneumonia. *Journal of Chemotherapy*.

[30] Aguilar P. R., Balsara K., Itoh A., Kollef M. H. (2016). A noteworthy case of acute bronchitis. *Annals of the American Thoracic Society*.

